# Comparison of Mean Postoperative Hemoglobin Concentrations in Patients Undergoing Total Knee Arthroplasty With Intravenous Versus Intraarticular Administration of Tranexamic Acid

**DOI:** 10.7759/cureus.68593

**Published:** 2024-09-03

**Authors:** Farhan Aslam, Hafiz Usman Arshad, Bilal Qammar, Izzah Shakeel, Zia Sidhu, Zunaira Shakeel, Hafiz Muhammad Arbaz, Tariq Rashid, Muhammad Ahsan Ishfaq, Muhammad Naveed Zafar, Mohsin Raza

**Affiliations:** 1 Trauma and Orthopedic Surgery Department, Sir Ganga Ram Hospital/Fatima Jinnah Medical University, Lahore, PAK; 2 Trauma and Orthopedic Surgery Department, Sir Ganga Ram Hospital, Lahore, PAK; 3 Trauma and Orthopedic Surgery Department, Shalamar Hospital, Lahore, PAK; 4 Medicine Department, Omer Hospital and Cardiac Center, Lahore, PAK; 5 Trauma and Orthopedic Surgery Department, Shalamar Hospital Lahore, Lahore, PAK; 6 Hematology Department, Sundas Foundation Hospital, Lahore, PAK; 7 General Surgery Department, Shalamar Hospital, Lahore, PAK; 8 Trauma and Orthopedic Surgery Department, Ghurki Hospital, Lahore, PAK; 9 General Surgery Department, Allied Hospital, Faisalabad, PAK

**Keywords:** postoperative hemoglobin, orthopedic surgery, joint replacement, total knee arthroplasty, tranexamic acid

## Abstract

Background: Total knee arthroplasty (TKA) may result in significant blood loss, but it is an effective and affordable treatment for severe osteoarthritis in the knees. While intravenous (IV) tranexamic acid (TXA) is a commonly used technique, intraarticular (IA) TXA has just recently started to gain traction in joint replacement procedures. The purpose of this research was to examine the mean postoperative hemoglobin concentration in order to assess the effectiveness of TXA administered IV vs IA after TKA.

Objective: To assess the effectiveness of intraarticular TXA against intravenous administration.

Materials and Methods: The six-month randomized controlled experiment was started from October 5, 2022, to April 4, 2023, at “the Orthopedics Department of Sir Ganga Ram Hospital in Lahore”. The experiment included 60 patients undergoing TKA, ranging in age from 30 to 70. All members of the surgical team, including the supervisor (a consultant surgeon), assistants, and researchers, were present throughout the surgery. A high, thigh tourniquet was employed in every case, and a medial parapatellar technique was performed as well. Before the tourniquet was inflated, individuals in the intravenous group received 1 g of TXA intravenously 15-30 minutes beforehand. In the IA group, the “patient received an injection of 2 g of TXA in a 20 mL solution” straight into the joint after the prosthesis was implanted and secured. Data were analyzed using SPSS (version 26), with numerical data (age, BMI, surgical length, and hemoglobin levels) presented as mean ± SD and categorical factors (gender, American Society of Anesthesiologists (ASA) class, anatomical side) shown as frequency and percentage. The mean postoperative hemoglobin levels were compared between groups using an independent sample t-test, with data stratified by various factors and p ≤ 0.05 considered significant.

Results: There were 60 patients in this study, ranging in age from 30 to 70. The mean±SD age was 48.73±10.35 years. Patients’ mean BMI was 25.51±4.48 kg/m², with representation across underweight, normal, overweight, and obese categories. The procedure took 173.10±32.61 minutes. The overall postoperative hemoglobin concentration was significantly higher in the IA TXA group (12.12±1.32 g/dL) compared to the IV TXA group (11.11±1.10 g/dL), with a p-value of 0.02. Additionally, when stratified by age, the IA TXA group consistently demonstrated higher postoperative hemoglobin levels across all age brackets, with significant differences observed in the 51-60 years (p = 0.001) and 61-70 years (p = 0.011) groups. Gender-based comparisons showed that IA TXA was associated with higher postoperative hemoglobin levels for both males (p < 0.05) and females (p < 0.05) compared to IV TXA.

Conclusion: This study demonstrates that IA administration of TXA is more effective in maintaining higher postoperative hemoglobin concentrations compared to IV TXA in patients undergoing TKA. The IA TXA group consistently achieved significantly higher hemoglobin levels across various age groups and both genders, indicating superior efficacy in reducing blood loss associated with TKA. These findings suggest that IA TXA could be a preferable alternative to IV TXA for enhancing postoperative hemoglobin recovery and potentially improving patient outcomes in knee arthroplasty procedures.

## Introduction

Blood loss during surgery is still a major issue, even though improvements in implant design and fixation have significantly raised functional outcomes and survival rates for total knee arthroplasty (TKA) [1]. A surgical incision is made during a TKA, which may need the patella to be dislocated in order to access the knee joint. Significant bleeding ensues from the disruption of soft tissue as a result [2]. Trauma from surgery during TKA may cause blood to leak or extravasate into the surrounding tissues. Because it is hidden, this blood loss is not as noticeable, but it contributes significantly to the total amount of blood loss. Damage to the surrounding tissue and disturbance of the blood vessels might result in extravasation [3, 4]. Even with intensive efforts to remove blood from the knee joint, blood may still remain in the intraarticular space. Concealed blood loss may result from the fact that some blood may not always be completely removed during surgery or the drainage process that follows [3, 4]. The average blood loss during TKA was reported to be 1518 mL [5] in research by Lotke et al. that calculated the perioperative reduction in hemoglobin levels. Operative blood loss and the ensuing requirement for blood transfusions raise the risk of anaphylactic and infectious complications and significantly postpone the patients’ objectives for physical therapy and rehabilitation [1, 6].

The significant breakthrough over the last 10 years, intraoperative attempts to reduce bleeding after TKA have included the use of TXA, a synthetic anti-fibrinolytic drug that prevents plasminogen from being activated to plasmin. It does not increase the likelihood of clot formation and has been used to reduce bleeding in patients undergoing TKA. It has the opposite effect and prevents a clot from dissolving [7]. The likelihood of deep vein thrombosis (DVT) is increased in these people due to the intravenous (IV) administration, however, [6, 7] a recent local research conducted by Furqan et al. [8] showed that the intraarticular (IA) injection of TXA was superior to the standard intravenous administration approach. This was because the IA administration was linked to a significantly higher mean postoperative hemoglobin concentration (11.09±0.39 vs. 9.93±1.73 g/dL p-value=0.03), which indicated less operative blood loss. Kornah et al. [9] have similarly documented a substantially higher mean postoperative hemoglobin concentration in the IA group (10.54±0.47 vs. 10.23±0.53 g/dL; p-value=0.004) in Egypt.

Numerous researchers have shown that TXA is effective in reducing bleeding during TKA, considerably decreasing the transfusion rate while not raising the risk of venous thromboembolism (VTE) [10-17]. Transplant drainage decreased statistically significantly (mean reduction of 290 mL) when tranexamic acid (TXA) was administered, according to a recent meta-analysis that looked at 19 trials including over 1100 patients. Blood transfer frequency decreased by 0.96 units, volume decreased by 440 mL, and total blood loss decreased by an average of 570 mL when TXA was used. The percentage of patients who needed transfusions of blood was drastically decreased by using TXA, with a relative probability of just 0.39. No statistically significant variations were seen among the three study groups for VTE or additional adverse events [18]. Several methods exist for administering TXA, including the oral, topical, and IV routes. Orthopedic surgeries have mostly made use of intravenous and topical techniques [12]. To be eligible for IV medicine, a patient must have normal renal clearance and be free of prior venous thromboembolic events or cardiac surgeries. Individuals for whom intravenous TXA is not an option may safely utilize topical TXA due to its low systemic absorption. The dose of TXA used in cardiac surgery is often ten times larger than that used in orthopedic surgery [13]. Three studies have looked at the efficacy of both topical and intravenous TXA. Both Gomez-Barrena et al. [12] and Patel et al. [15] demonstrated that the rate of transfusion reduction was not affected by whether the TXA was applied topically or administered intravenously. Seo et al. [16] found that topical TXA was somewhat more efficacious than IV TXA, resulting in a mean decrease of 426 mL of bleeding compared to 528 mL. The findings showed that compared to IV treatment, TXA injection significantly decreased bleeding and transfusion needs.

When used in conjunction with TKA, TXA has the potential to enhance patient well-being. This is accomplished by encouraging hemodynamic stability, hastening the healing process, and lowering the risk of complications and problems connected to transfusions. Both IV and topical TXA have been recommended by some clinics [19, 20]. TXA has been shown to be very cost-effective, with a cheap cost, an easy-to-follow dosage schedule, and a significant impact on the need for transfusions after TKA [21]. This research suggests that IA application of TXA is preferable to the traditional intravenous administration approach, which calls for a modification of existing therapeutic practice. Before drawing any conclusions, it’s important to note that the available data is inconclusive. Amin et al. [22] found no significant differences in the groups’ mean postoperative hemoglobin levels (9.93±1.14 vs. 9.87±1.26 g/dL: p-value=0.724) in another local research. Uzer et al. [23] likewise found a similar negligible difference in Turkey (10.9+1.3 vs. 11±1.6 g/dL; p-value = 0.541). The IA method was shown to be inferior to the intravenous route by Karaduman [24], and this resulted in a substantially lower postoperative hemoglobin concentration (11.09±1.61 vs. 12.42±1.07 g/dL; p-value=0.002). This further complicates the problem.

Therefore, the available data was equivocal; some research asserted that the mean postoperative hemoglobin concentration was equivalent for both methods [22, 23], while others stated that the IA route was superior [8, 9], and yet others claimed that the intravenous approach was better [24]. A plausible rationale for the discrepancy among studies might be the absence of research on impact modifiers such as patient age, gender, BMI, and surgical length. We replicated this experiment to better corroborate the findings in light of the dispute in the previously published literature, and we stratified the data to address these impact modifiers. In the future, orthopedic practitioners will be able to choose a more suitable method of administering TXA to patients undergoing total knee replacements based on the findings of this research.

Objective

The study objective is to evaluate the mean postoperative hemoglobin concentration in patients having total knee replacements with TXA administered intravenously vs intraarticularly.

## Materials and methods

Study design

Using non-probability sequential sampling, the research was conducted as a “randomized controlled trial at the Department of Orthopedic Surgery, Sir Ganga Ram Hospital, Lahore”, during a 6-month period, from September 5, 2022, to April 4, 2023.

Sample size

With “80% test power and a 95% confidence interval, a sample size of 60 patients (30 cases in each group) is determined”. The predicted “mean postoperative hemoglobin” concentration is assumed to be 11.09±0.39 g/dL with IA TXA and 9.93±1.73 g/dL with intravenous TXA [8].

Sample selection

Using non-probability sequential sampling, the research was conducted as a randomized controlled trial at the Department of Orthopedic Surgery, Sir Ganga Ram Hospital, Lahore, during a 6-month period, from September 5, 2022, to April 4, 2023. The study included adult patients of both genders between the ages of 30 and 70 undergoing TKA, as defined by the operative criteria. The cohort predominantly consisted of patients under the age of 50, with primary etiologies including post-traumatic arthritis, inflammatory arthritis (such as rheumatoid arthritis), and secondary osteoarthritis due to previous knee injuries or congenital deformities. Patients undergoing revision arthroplasty and those with comorbidities documented in clinical records that the American Society of Anesthesiologists (ASA) classified as class III or above were not included. Additionally, patients with abnormal pre-operative examination results in abnormal coagulation profiles (INR ≥2.5), renal function (serum creatinine ≥1.2 mg/dL), or liver function (serum bilirubin ≥1.2 mg/dL) were excluded.

Data collection procedure

Sixty patients (30 patients in each group) who underwent TKA on the orthopedic department’s elective lists of Sir Ganga Ram Hospital, Lahore, and met the aforementioned requirements were counseled and given an explanation of the study after receiving approval from the hospital’s ethical review board. Every patient provided their written informed permission and provided a comprehensive medical history. The patients were then randomly assigned to one of two groups: Group A, which consisted of 30 people, got TXA intravenously, while Group B, also consisting of 30 people, received TXA intraarticularly.

The randomization process was conducted using a computer-generated randomization list, which ensured equal chances of assignment to either the IV or IA TXA groups. To maintain allocation concealment, assignments were placed in sequentially numbered, opaque, sealed envelopes. Upon obtaining informed consent, a member of the research team not involved in clinical management opened the next envelope in the sequence, assigning the patient to the indicated group. The randomization was single-blind, with patients unaware of their group assignment, and the surgeon aware of correct TXA administration but not involved in outcome assessments to maintain objectivity. This method minimized selection bias and ensured comparable baseline characteristics between groups.

A high thigh tourniquet was employed in every case, and a medial parapatellar approach was performed as well. The patella was resurfaced and the tibial and femoral components were cemented using a posterior stabilized knee prosthesis (Zimmer, Ltd. UK) that sacrifices the posterior cruciate ligament. Spinal anesthesia was used to perform the procedures. Establishing hemostasis and deflating the tourniquet were prerequisites to closing the wound. A drain was not put in. A compression bandage was used with the knee bent for the first 24 hours after surgery. Before the tourniquet was inflated, individuals in the intravenous category received 1 g of TXA intravenously 15-30 minutes beforehand. In the IA group, patients received an injection of 2 g of TXA in a 20 mL solution straight into the joint after the prosthesis was implanted and secured. To ensure objectivity, a single resident (the candidate) from a single lab (a hospital lab that patients were not charged for) was responsible for all patients’ hemoglobin assessments, pre- and post-procedure care. The consultant performed all procedures alongside the candidate, and he had over 15 years of experience. By exclusion, confounding factors were kept under control. The applicant presented a pre-designed proforma on which postoperative hemoglobin concentrations and patient demographic information were recorded (see Appendices).

The study utilized the ASA physical status classification system to categorize patients preoperatively. The inclusion criteria were limited to patients classified as ASA class I or II, which denotes individuals who are either healthy (class I) or have mild systemic disease without substantial functional limitations (class II). This classification ensures a standard evaluation of patient fitness before surgery, aiding in the assessment of surgical risk and anesthesia management. The ASA classification system is widely recognized and used globally, including by the Southeast Asian region, for pre-operative evaluation.

Data analysis procedure

All of the gathered data was examined using the most recent version of SPSS (26). The mean ±SD was used to display numerical data such as age, BMI, surgical length, and hemoglobin levels before and after surgery. “The mean postoperative hemoglobin levels were compared between the groups using an independent sample t-test, with p ≤0.05 being considered significant.” The frequency and percentage of categorical factors, such as gender, ASA class (I/II), and anatomical side (right/left), were shown. To account for effect modifiers, the data was stratified by age, gender, BMI, ASA class (I/II), length of operation, and anatomical side (left/right). An independent sample t-test was used after stratification, with p < 0.05 considered significant.

Ethical consideration

The ethical review committee of the College of Physicians and Surgeons Pakistan (CPSP) approved the study, which was carried out in compliance with ethical guidelines, under reference number CPSP/REU/OSG-2021-059-2658, dated September 15, 2022. Every participant gave their informed agreement, guaranteeing their free choice to participate and knowledge of the goals and methods of the study. The research followed patient rights and confidentiality guidelines all along the trial.

## Results

Sixty patients were included in this study, divided into two groups: the IV TXA group (n = 30) and the IA TXA group (n = 30). Patients’ ages ranged from 30 to 70 years, with a mean age of 48.73 ± 10.35 years. The gender distribution was balanced in both groups, with 15 males (50%) and 15 females (50%) in each group. In terms of ASA classification, 15 patients (50%) in the IV TXA group and 14 patients (46.67%) in the IA TXA group were classified as ASA Class I, while 15 patients (50%) in the IV TXA group and 16 patients (53.33%) in the IA TXA group were classified as ASA Class II, indicating a predominance of mild to moderate systemic disease.

The average BMI for the cohort was 24.5 ± 3.2 kg/m², consistent across both groups. The mean duration of surgery was 155 ± 28 minutes. Regarding the anatomical side of the surgery, 16 patients (53.33%) in the IV TXA group and 15 patients (50%) in the IA TXA group underwent surgery on the right knee, while 14 patients (46.67%) in the IV TXA group and 15 patients (50%) in the IA TXA group had surgery on the left knee.

Pre-operative hemoglobin levels averaged 13.69 ± 1.20 g/dL, with a mean of 13.5 ± 1.17 g/dL in the IV TXA group and 13.88 ± 1.22 g/dL in the IA TXA group. Postoperative hemoglobin levels averaged 11.61 ± 1.31 g/dL, with 11.11 ± 1.10 g/dL in the IV TXA group and 12.12 ± 1.32 g/dL in the IA TXA group (Table 1).

**Table 1:** Demographic and Clinical Characteristics of Patients Undergoing Total Knee Arthroplasty (TKA) ASA class: American Society of Anesthesiologists Physical Status Classification; BMI: body mass index; IV: intravenous; IA: intraarticular; TXA: tranexamic acid

Variable		IV TXA Group (n = 30)	IA TXA Group (n = 30)	Total (N = 60)
Age (years)	Mean ± SD	48.73 ± 10.35	48.73 ± 10.35	48.73 ± 10.35
Range		30 - 69	30 - 69	30 - 69
Gender	Male	15 (50%)	15 (50%)	30 (50%)
	Female	15 (50%)	15 (50%)	30 (50%)
ASA Class	Class I	15 (50%)	14 (46.67%)	29 (48.33%)
	Class II	15 (50%)	16 (53.33%)	31 (51.67%)
BMI (kg/m²)	Mean ± SD	24.5 ± 3.2	24.5 ± 3.2	24.5 ± 3.2
Duration of Surgery (min)	Mean ± SD	155 ± 28	155 ± 28	155 ± 28
Anatomical Side of Surgery	Right	16 (53.33%)	15 (50%)	31 (51.67%)
	Left	14 (46.67%)	15 (50%)	29 (48.33%)
Pre-Operative Hemoglobin (g/dL)	Mean ± SD	13.5 ± 1.17	13.88 ± 1.22	13.69 ± 1.20
Postoperative Hemoglobin (g/dL)	Mean ± SD	11.11 ± 1.10	12.12 ± 1.32	11.61 ± 1.31

To compare the mean Hb concentrations between the IV and IA TXA groups, an independent sample t-test was utilized. The results showed that the IA TXA group had a significantly higher mean postoperative Hb concentration compared to the IV TXA group. The mean pre-operative Hb concentration for the IV TXA group was 13.5 ± 1.17 g/dL, and the postoperative Hb concentration was 11.11 ± 1.10 g/dL. For the IA TXA group, the mean pre-operative Hb concentration was 13.88 ± 1.22 g/dL, and the postoperative Hb concentration was 12.12 ± 1.32 g/dL. Significant differences were noted overall with a p-value of 0.02 for the postoperative Hb. The comparison within specific age groups revealed no significant disparities between the IV and IA TXA groups in the 30-40 and 41-50 years age ranges. Significant differences were observed in the 51-60 (p = 0.001) and 61-70 (p = 0.011)-year-age groups. Additionally, postoperative Hb levels differed significantly between genders, with males in the IV TXA group having a mean Hb of 10.62 ± 0.75 g/dL and females having 11.32 ± 1.24 g/dL, compared to IA TXA males at 11.97 ± 1.28 g/dL and females at 12.26 ± 1.36 g/dL. A significance level of ≤0.05 was used to determine statistical significance (Table 2).

**Table 2:** Comprehensive Comparison of Hemoglobin Levels Across Different Groups, Stratified by Age, Gender, and Pre-operative/Postoperative Status p-value <0.05 was significant.

Category	Group		N	Mean Hb (g/dL)	Standard Deviation	P-value
Pre-operative Hb	IV TXA		30	13.5	1.1655	0.2
	IA TXA		30	13.88	1.2199	
Overall Postoperative Hb	IV TXA		30	11.11	1.0997	0.02
	IA TXA		30	12.12	1.3244	
Postoperative Hb by Age Group	Age 30-40	IV TXA	5	11.22	0.92	0.422
		IA TXA	9	11.95	1.82	
	Age 41-50	IV TXA	11	11.35	1.27	0.532
		IA TXA	8	11.71	1.12	
	Age 51-60	IV TXA	8	10.77	0.74	0.001
		IA TXA	10	12.4	0.99	
	Age 61-70	IV TXA	6	10.46	1.18	0.011
		IA TXA	3	12.96	0.48	
Postoperative Hb by Gender	Male	IV TXA	14	10.62	0.75	<0.05
		IA TXA	15	11.97	1.28	
	Female	IV TXA	16	11.32	1.24	<0.05
		IA TXA	15	12.26	1.36	

The findings showed that, in patients with normal BMI, the mean postoperative Hb of the two groups did not vary statistically significantly. However, there was a big difference between the two groups in individuals with a BMI of overweight and obese, with IA TXA being better than IV TXA. P -values less than 0.05 were considered significant (Table 3).

**Table 3:** Comparison of Mean Postoperative Hemoglobin Levels, Stratified by BMI (Independent sample T-test) p-value <0.05 was significant. Only one patient was in the underweight group, hence mean difference in post-op Hb could not be calculated.

BMI Range		Group	N	Mean	Std. Deviation	P-value
Normal (18.5 – 24.9 kg/m^2^)	Post-op HB (g/dL)	IV TXA	10	11.675	1.1316	0.858
		IA TXA	16	11.769	1.3809	
Overweight (25 – 29.9 kg/m^2^)	Post-op HB (g/dL)	IV TXA	13	10.735	.8236	<0.001
		IA TXA	8	12.769	1.1646	
Obese (30 – 34.9 kg/m^2^)	Post-op HB (g/dL)	IV TXA	7	10.509	1.1034	0.030
		IA TXA	5	12.069	.9854	

The findings demonstrated that while the postoperative hemoglobin concentrations of patients in ASA Class I were better in the IA TXA group compared to the IV TXA group, the differences were statistically insignificant. In contrast, they were in ASA Class II statistically significant. A p-value of less than 0.05 was deemed significant (Table 4).

**Table 4:** Comparison of Mean Postoperative Hemoglobin Levels, Stratified by the ASA class ASA class: American Society of Anesthesiologists Physical Status Classification, p-value <0.05 was significant.

Independent sample T-test						
ASA class		Group	N	Mean	Std. Deviation	P-value
ASA-I	Post-op Hb (g/dL)	IV TXA	14	11.296	1.1832	0.118
		IA TXA	15	12.121	1.5292	
ASA-II	Post-op Hb (g/dL)	IV TXA	16	10.732	.9503	<0.001
		IA TXA	15	12.158	1.0748	

**Figure 1:**
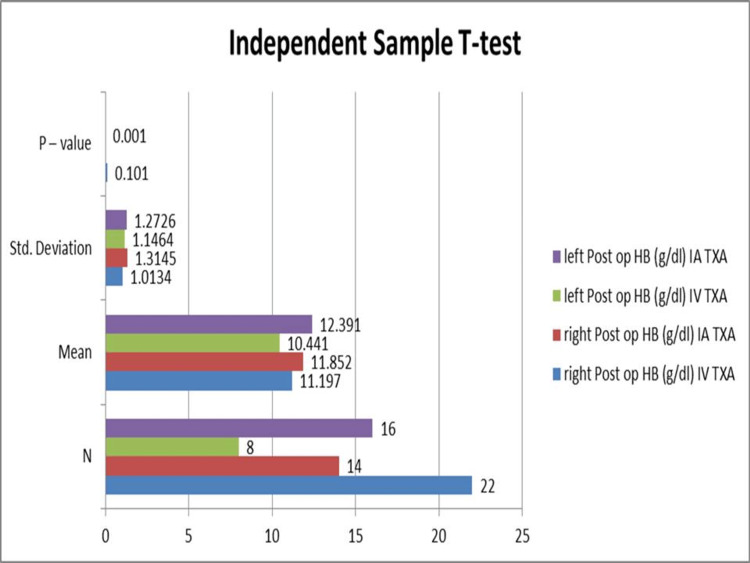
Comparison of mean postoperative hemoglobin levels, stratified by anatomical site

Although there was a statistically insignificant difference between the IA TXA and IV TXA groups in terms of postoperative Hb concentration, the data demonstrated that the IA TXA group had a higher concentration of hemoglobin than the IV TXA group. Statistical significance was seen in patients who had left-sided TKA. A p-value of less than or equal to 0.05 was considered notable, as illustrated in Figure 1.

The findings demonstrated that the disparities in postoperative Hb concentration between the two groups of patients receiving TKA were larger as the time of surgery increased. For patients whose surgeries lasted between 121 and 180 minutes, the average postoperative HB (g/dL) of intravenous TXA was 10.921±0.9788 g/dL, whereas that of intramuscular TXA was 11.9731±0.3183 g/dL. Patients whose operation lasted between 181 and 240 minutes had an average postoperative HB (g/dL) of 11.145±1.3158 g/dL with IV TXA and 12.389±1.2838 g/dL with IA TXA (Table 5).

**Table 5:** Comparison of Mean Postoperative Hemoglobin Levels, Stratified by Duration of Surgery p-value <0.05 was significant.

Duration of Surgery (min)	Group	N	Mean	Std. Deviation	P-value
121-180	IV TXA	20	10.921	0.9788	0.008
121-180	IA TXA	18	11.973	1.3183	
181-240	IV TXA	10	11.145	1.3158	0.037
181-240	IA TXA	12	12.389	1.2838	

## Discussion

An orthopedic operation called TKA is often performed to help people with osteoarthritis in their knees live better lives. There is still worry about blood loss during and after TKA, hence several strategies are used to reduce the postoperative Hb decline. One such technique for reducing bleeding is the IV or IA infusion of TXA. As an antifibrinolytic medication, TXA, a synthetic version of the amino acid lysine, works by competitively inhibiting the lysine-binding sites in the plasmin & plasminogen activator molecules, preventing the dissolution of the fibrin clot [25]. Although TXA either IV or IA has been shown to be effective in minimizing bleeding during TKA, the best way to administer the medication has been debated. The purpose of this research was to evaluate the impact of IV vs IA TXA on transfusion rates, blood loss during surgery, and edema of the lower limbs after TKA. The purpose of this research was to examine how these two delivery methods affect the Hb concentration after surgery.

There were 60 patients in this research, ranging in age, gender, BMI, ASA class, and surgical variables. Not a single patient was missed for follow-up. Hemoglobin concentrations after surgery were measured and examined in connection with patient attributes. In our research, the average age of the patients was 48.73±10.346 years. The mean age of the patients in an Indian RCT was found to be 58.77 ± 10.14 years [26]. In contrast, the mean age of patients in another study by Subramanyam et al. was 62.9 ± 6.8 years [27]. In our study, the mean BMI of patients was 25.51±4.48 kg/m^2^. This is consistent with a study by Aggarwal et al. in which the mean BMI of patients was 26.33 ± 3.79 kg/m^2^ [26]. Similarly, Subramanyam et al. had a mean BMI of 28.9 kg/m^2^ [27].

In our study, patients with normal BMI did not exhibit a significant difference between IA and IV TXA, but those in the overweight and obese categories had superior outcomes with IA TXA. This finding may be related to the higher adipose tissue content in overweight and obese patients, which could increase bleeding risk during surgery and require a more localized TXA effect provided by IA administration. No previous studies regarding the comparison of IA TXA vs IV TXA with respect to BMI on blood loss during TKA have been found. Further research is warranted in this aspect. In our study, 1 g of TXA was injected intravenously into the patients in the intravenous group, and the injection was given 15-30 minutes prior to the tourniquet being inflated. When the prosthesis was placed and fixed, patients in the IA group were given an injection of 2 g of TXA in a 20 mL solution directly into the joint. The majority of earlier research used 1 g of TXA in the IV group and 3 g of TXA in the IA group, either after closure or cementation [28, 29].

In contrast, several trials used 1.5-3 g of TXA in the IA group and 10-20 mg in the IV group, either before or after cementation and tourniquet release [30]. Compared to IV TXA, the mean postoperative Hb level for IA TXA was superior. With a p-value of ≤0.05 being considered significant, IV TXA had a mean postoperative Hb concentration of 11.105±1.09 g/dL and IA TXA had a mean postoperative concentration of 12.116±1.324 g/dL. Aggarwal et al.’s [26] research revealed a statistically significant (P <0.001) difference in postoperative hemoglobin (9.66 ± 1.47/10.30 ± 1.11 gm/dL), with IV TXA seeing a greater decline in hemoglobin than the IA TXA group (2.69 ± 1.16/ 1.60 ± 0.68 gm/dL) [26]. In our investigation, there was a higher difference in postoperative Hb concentration between IA and IV TXA in patients who had lengthier surgeries. This outcome is most likely the consequence of prolonged exposure to blood during lengthy surgeries as well as surgical stress. Because of its confined activity, IA TXA seems to work better in these situations. Further study is required on these and related topics.

Limitations and future suggestions

The present research has some notable constraints. The research is deficient in a control group that would have received either IV or IA saline, which would have been crucial for making meaningful comparisons. The sample size of this research is somewhat small, which might restrict the applicability of the findings. Conducting research on a greater scale would provide more reliable and strong data. The trial was done in a solitary location, perhaps leading to bias in choosing patients and surgical procedures. TXA theoretically poses a thrombosis risk, however this has not been clinically substantiated. Our research did not show any higher risks of DVT or pulmonary embolism (PE). Our research did not evaluate long-term effects, such as comorbidities and functional recovery. While the research made efforts to manage surgical variables, there is always a possibility of variances in surgical technique and perioperative care that might impact postoperative Hb levels.

## Conclusions

The study assessed the effectiveness of IV versus IA TXA administration in patients undergoing first-time TKA. Conducted over six months with 60 patients, the study revealed that IA TXA significantly improved postoperative Hb concentrations compared to IV TXA (p < 0.05). This suggests that IA TXA is more effective in preserving postoperative Hb levels during TKA. These findings emphasize the potential benefits of IA TXA for orthopedic surgeons and highlight the need for further research with larger sample sizes to confirm these results and evaluate long-term outcomes and potential complications.

## Figures and Tables

**Figure 2:**
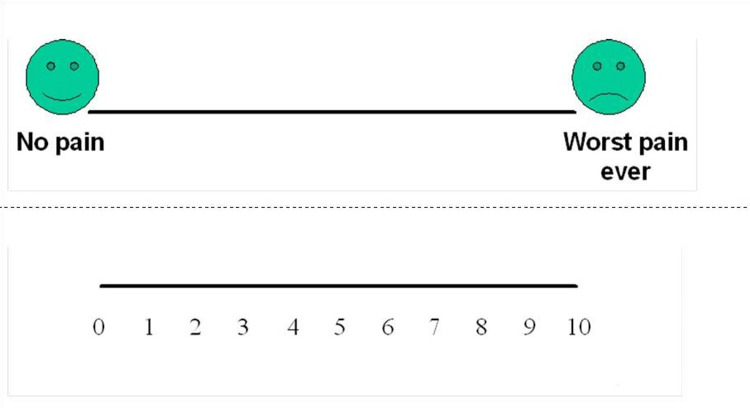
Visual Analog Scale

## Appendices

Data collection instrument proforma

Comparison of Mean Postoperative Hemoglobin Concentration in Patients undergoing Total Knee Arthroplasty with Intravenous versus Intraarticular administration of Tranexamic Acid

*Case No*                

Name of Patient: ____________________________

S/D/W/O: _________________________________

Age: _____________________________________ years

*Gender*                      

Male                           

Female

Anatomical Side

Right

Left

ASA Class

ASA-I

ASA-II

BMI: ________________________________ kg/m^2^          

Duration of Surgery: ____________________ minutes

Study Group:                                                                             A                                  B                                                                                                                                         

Pre-Operative hemoglobin: ________________________________ g/dL

Postoperative hemoglobin: ________________________________ g/dL

Appendix-I: Visual analog scale (VAS)

The following diagram were printed on an A4 sheet ensuring that the lines were exactly 10 cm in length.

The printout were then folded at the dotted line.

Patient were asked to mark the line according to his/ her pain by moving from no pain to worst pain.

Patients were not shown the numbered side.

After that the VAS score were measured by unfolding the numbered side and recording the corresponding score.

Appendix-II: American Society of Anesthesiologists (ASA) Classification

ASA Physical Status 1 - A normal healthy patient

ASA Physical Status 2 - A patient with mild systemic disease

ASA Physical Status 3 - A patient with severe systemic disease

ASA Physical Status 4 - A patient with severe systemic disease that is a constant threat to life

ASA Physical Status 5 - A moribund patient who is not expected to survive without the operation

ASA Physical Status 6 - A declared brain-dead patient whose organs are being removed for donor purposes
